# Lack of Truth-Telling in Palliative Care and Its Effects among Nurses and Nursing Students

**DOI:** 10.3390/bs10050088

**Published:** 2020-05-11

**Authors:** Ines Testoni, Michael Alexander Wieser, Dafni Kapelis, Sara Pompele, Marino Bonaventura, Robert Crupi

**Affiliations:** 1 Department of Philosophy, Sociology, Pedagogy and Applied psychology (FISPPA), University of Padova, 35131 Padova, Italy; ines.testoni@unipd.it (I.T.); sara.pompele93@gmail.com (S.P.); 2 Arts Therapies Research Center, University of Haifa, Haifa 3498838, Israel; 3 Institute of Psychology, University of Klagenfurt, 9020 Klagenfurt, Austria; 4 Palliative Care Department, ULSS n. 8 Asolo, 31011 Treviso, Italy; dafni.kapelis@gmail.com; 5 Department of Neurosciences, University of Padova, 35128 Padova, Italy; marino.bonaventura@unipd.it; 6 NewYork-Presbyterian Queens, New York, NY 11355, USA; rcrupi54@gmail.com

**Keywords:** truth-telling, attitudes, palliative care, grief, euthanasia, moral distress

## Abstract

Unclear communication of inauspicious prognoses may disorientate both patients and their relatives, drastically jeopardizing the planning of palliative care. This paper considers the issue of truth-telling in the communicative problems of nurses and students of nursing with terminally ill patients. The fundamental objective is the analysis of the difficulties related to the lack of truth-telling and how it might impact their professional and personal lives. A qualitative study was realized, involving 47 participants, both nurses (25) and nursing students (22), working in palliative care units or in associations of volunteers for the assistance of oncological patients. The exploration was focused on the way they relate to patients who are not aware of their real health conditions and their consequences. Particular attention was paid to their opinions concerning what could be done in order to manage such problematic situations in the near future.

## 1. Introduction

Giving information to a patient concerning his/her health condition, especially when this means delivering some bad news, is still a very debated aspect in the field of medical ethics, and it is an issue linked to the complexity of human, juridical, and ethical matters.

Up until the second half of the 20th century, there was a deeply held belief that it was necessary, in order to make a patient more compliant and not to cause him/her too much emotional distress (which was believed could lead to a faster worsening of his/her condition), not to disclose a ‘bad’ diagnosis or poor prognosis, or that, at least, such a disclosure should be withheld as long as possible [1].

This view still persists, but has gradually faded as the idea has taken hold that an honest communication with the patient, a process called truth-telling, is essential for the patient to grow stronger in their outlook. Truth-telling, therefore, as regards the medical field, could be defined as a total openness concerning diagnosis and prognosis [2], and it generally refers to the disclosure of bad news [3], defined as any information that drastically changes the recipient’s present and future life expectations [4].

However, being sincere in the communication with a patient still appears to be extremely complicated for healthcare professionals. As a result, the patient is often excluded and not an active participant in his/her own treatment. In everyday clinical practice, when healthcare professionals realize that a patient lacks a real understanding of his/her condition, they can experience significant distress because of their difficulty in managing these situations. In particular, they may struggle with the uncertainty of how to best approach the patient. 

Among healthcare professionals, nurses spend the largest amount of time with patients, especially those admitted to cancer wards or hospices. For this reason, nurses are more intimately involved with their patients and may encounter difficulties around the issue of truth-telling, especially when there has not been an honest communication between the patient and their physician(s) or family. Nurses themselves identify these situations as a particularly challenging aspect of their job [5].

The aim of this research was therefore to further explore the experience lived by nurses working in palliative care units, assessing in particular the ways in which they relate to patients who are not aware of their real health conditions and their implications. The research was conducted following a qualitative methodology, through semi-structured interviews submitted to 47 participants, both nurses and nursing students. This article will present a literature review concerning truth-telling, information regarding the main objectives of the research, the methodology, the participants, and the way the collected data were analyzed, as well as the main results and their discussion.

### Literature Review

Truth-telling, as regards the medical field, can be defined as a total openness concerning diagnosis and prognosis [2], and it generally refers to the disclosure of bad news [3], defined as any information that drastically changes the recipient’s present and future life expectations [4].

In Europe, the patients’ right to receive a full and complete disclosure concerning their condition is affirmed by the Oviedo Convention (1997) [6], and, in Italy, by the recent Law n. 219 of the 22nd of December 2017 entitled “Norms concerning informed consent and the availability of advanced treatments” (Norme in materia di consenso informato e di disposizioni anticipate di trattamento), which states every individual’s right to be fully informed concerning his/her health condition as well as to freely choose which treatments to accept or to refuse. Furthermore, in the same country, the Code of Medical Deontology (2014) [7] and the Nurse’s Deontological Code (2009) [8] highlight the importance of listening to patients’ needs and desires and their right to always be informed and properly supported.

Even still, issues inherent to the breaking of bad news are still debated because they are linked to juridical and ethical matters, as well as the lingering 20th century belief, widely shared all over the world, that it is better to conceal from patients their real health conditions [1]. In particular, this outdated approach is still practiced with terminally ill patients as much in Italy as in other European countries [2,3,4,5,6,7,8,9], since doctors with low relational skills fear worsening the level of suffering for their patients [9,10]. Unfortunately, physicians’ difficulties in communicating bad news may disorientate both patients and their relatives, severely jeopardizing the planning of palliative care [11]. There is no evidence supporting the idea that concealing the truth helps patients feel better, while, on the contrary, research underscores high levels of anxiety and depression among patients who are not correctly informed with respect to their prognoses [10]. In fact, it has been shown that sincere communication with patients, especially with those who are seriously ill and have an unfavorable prognosis, is beneficial to the patients’ psychological adaptation, coping, and satisfaction concerning treatments [12]. Moreover, the majority of patients, even when seriously ill, want to know the truth concerning their condition as well [13]. At the same time, for a large number of professional caregivers, truth-telling is considered crucial to recognizing and satisfying families’ needs [11] while coping with ethical obligations [2].

Without a full understanding of their prognoses and the risks and benefits of each treatment, patients cannot make mindful decisions concerning their plan of care to avoid what they feel to be useless or aggressive treatments [10]. Complicating this matter, the lack of coping skills of relatives may create further communicative problems for physicians. In fact, even when the healthcare professionals are actually ready to tell patients the truth, their family members often oppose this therapeutic phase, and therefore become an obstacle to truth-telling [14,15]. Literature has already considered how unclear communications between patients, physicians, and nurses can contribute to the development of burnout, as well as to high levels of stress and work turnover among healthcare professionals [16].

Nurses work in the communicative area between physicians and patients, and spend the largest amount of time with the latter. They are the healthcare category that most intensely suffers from the effects of lack of truth-telling because they are not allowed to communicate any prognoses [17]. Unfortunately, literature on the effect of this aspect is quite scarce, and, in particular, there is a lack of studies on their preoccupations caused by doubts concerning the awareness of the patient and difficulties in answering their prognosis questions. Indeed, nurses’ stress oscillates between the fear of destroying hope when there is no longer remedy and giving hope when there are unrealistic fears, and often they do not feel capable of dealing with such communicative problems [14]. All of this makes their emotional state and the burden of professional fatigue much worse [18]. The dilemmas that nurses have to deal with in end-of-life patients are particularly distressing when there is a conflict between their idea of what is right and the different opinions of other members of the medical team or the patient’s family, so that their desires are in contrast with their professional standards and main directives [11]. These antinomies cause feelings of impotence, inferiority, frustration, and rage toward both the patient’s family and other colleagues [13]. The present study wanted to consider all of these communicative problems and the point of view of nurses and students of nursing who work with terminally ill patients.

## 2. Material and Methods

### 2.1. Objectives

The aim of this investigation is to explore the experiences of nurses and students of nursing working in palliative care units. The fundamental objective is the analysis of the difficulties related to the lack of truth-telling and how it might impact their professional and personal lives. In particular, this study explored all of those situations in which the nurses had to relate to patients who were not fully aware of their real health conditions. Particular attention was paid to their opinions concerning what could be done in order to manage such problematic situations in the near future. The research followed the American Psychological Association (APA)’s Ethical Principles of Psychologists and Code of Conduct and the principles of the Declaration of Helsinki, obtaining the approval of the Padova University Ethics Committee for Experimentation. The ethical code for this research is B507F11B16DC9C80A16A8C72F60C8EA7.

### 2.2. Participants

The survey involved 47 participants, among whom 25 were professional nurses (53%) and 22 were nursing students (47%). Of these, 38 of them were female (81%) and 9 of them were male (19%). The mean age of all participants was 31 years, and, specifically for nursing students and professional nurses, it was 23 years (SD 1.36), and 39 years (SD 13.7), respectively. The professional nurses’ mean length of service was 13 years (SD 12.60), while their mean length of service in palliative care was 5.5 years (SD 5) (Table 1). The nurses worked either in one of two hospices or in associations of volunteers for assistance in end-of-life care, while nursing students were from a major university in the same area. All participants were informed of the research methodology and its objectives and signed the informed consent. Then, they were asked to fill in a questionnaire concerning their personal data, such as their gender, age, nationality, professional training, length of service, and number of years of service in palliative care units. In order to guarantee their privacy, every interview was identified with a number, which could not be traced back to the participants’ identities.

### 2.3. Methodology

The survey was realized through the Interpretative Phenomenological Analysis (IPA) because it makes it easier to understand biographical stories in ethnographic healthcare research [19,20]. In order to get as close as possible to the participants’ perspective, the IPA follows two processes: People are asked to make sense of their experiences through a detailed narration guided by the researcher; the researcher then tries to gain insight into how the participant, in a given context, arrives at their understanding. Through this method, the researchers get as close as they can to the point of view of the person who experiences a certain phenomenon [21,22]. The interviewer shadowed a grid of topics useful in supporting the dialogues. Some examples of questions are the following: “Have you ever not been completely honest with one of your patients concerning his/her clinical condition, or have you ever been somehow involved in a situation in which someone else was not completely honest with a patient? Why did that happen?”; “How did you feel about that? Did that (not being completely honest with a patient) represent a problem or a burden for you? If not, why not?”; “Do you believe that not being sincere with a patient concerning his/her clinical condition is the most appropriate choice?”; “In your opinion, which strategy could be the best one in order to understand the situation of patients?”; “How do you manage the omission of truth or the provision of the partial truth in the relationship with the patient?” The interviews lasted about 30 minutes each and were conducted in places chosen by participants in order to make them feel comfortable.

The work of the interviewer was constantly supervised by a female psychologist, professor, and psychotherapist. The answers were tape-recorded and transcribed verbatim, obtaining the corpora, which were analyzed using the framework method for thematic qualitative analysis and to examine the narrations in terms of their principal concepts at the basis of the discourses [23,24]. Two researchers processed the narrations through an analysis based on both prior categories and categories that only became clear as work proceeded [25,26]. The former were the basic predetermined themes from which the latter emerged as unexpected topics. The phases were as follows: Creation of the corpora and textual analysis; preparatory organization of the texts; generation of categories or themes; coding data; understanding the prototypical phrases; searching for alternative explanations; selection of the relationships between the main categories; discussion, writing up of the report, and construction of the diagrams [27]. The textual analysis was performed by highlighting recurrent themes that appeared to be particularly meaningful for each participant. Shared themes found in the other participants’ narrations were then grouped together to form broader distinct categories [28]. The analyses of texts were conducted according to three authors of the research (the interviewer, the supervisor, and another male professor). They first analyzed the texts individually; then, they considered the main different interpretations and decided on the final one, quite like the Delphi method process [29]. Data were collected in 2018, and the analysis was performed with the software *Atlas.ti.*

## 3. Results

From the data analysis, four areas of thematic prevalence emerged: “The impairment of the relationship with the patient”; “relatives and the conspiracy of silence”; “ethical dilemmas and emotional burden”; and “absence of a bridge between active treatments and palliative care”.

### 3.1. First Area of Thematic Prevalence: The Impairment of the Relationship with the Patient

Participants reported that lack of sincerity in communication with a patient inevitably impairs the relationship with him/her because it becomes rigid, more detached, and loses spontaneity, as highlighted by Stella (nursing student, 24 years old): *“Not being able to be spontaneous makes me appear fake in what I say to the patient, and I think the patient is aware of this”.*

Such a rigid attitude results from the nurse’s fear of disclosing some delicate information the patient does not yet know, as highlighted by Denise (professional nurse, 35 years old): *“With a patient who is not aware of his/her condition, you are always careful, since you never really know how he/she will interpret your words, so you always need to pay attention to what you say and do. It is really stressful*”.

The difficulty in dealing with these situations often brings the nurse to divert communication toward superficial or inessential issues: *“We try to talk about something different that is not their clinical condition, and if the patient is prone to asking many questions, we try to speak a little less, hoping certain questions will not be asked”*, said Jennifer (nursing student, 22 years old). Sometimes, nurses drastically try to avoid communication with patients, especially when the questions become too clear, insistent, and direct: *“We cannot even enter the room, you know. And then, because you cannot be false, you avoid the patient’s gaze, which is not a nice thing to do”,* as Rosa (professional nurse, 45 years old) expressed. *“I put a barrier between me and the patient because I felt almost embarrassed to do things without her consent”*, stated Lucia (nursing student, 22 years old).

Sometimes, they try to solve the problem with physicians, but without results, as Laura (professional nurse, 24 years old) reported: *“I spoke with the doctor, and I told him that I could not do this anymore. I explained that I was not able to enter a patient’s room and pretend. The doctors see another fifty patients for fifteen minutes each, while I am the one who stays with them all day. However, nothing changed”.*

The lack of spontaneity toward the patient is perceived as an extremely important element that contributes to further deteriorating the nurse–patient relationship because when the patient becomes aware of the lack of sincerity, he/she feels gravely betrayed: *“If he becomes aware you lied to him, I believe he loses his trust in you too”,* said Rosa. A similar example is described by Giovanni (nursing student, 22 years old): *“The patient learned that he was going to die after I had been working with him for a while. I only took care of him for two weeks, but my supervisor had an appointment with him every day, for another four months. I understood that the insincere communication of my supervisor with the patient compromised the quality of both our relationships with the patient”*. When the doctor has not properly communicated the prognosis, many difficulties do arise, and the only strategy considered appropriate and better than the avoidance of the relationship is to guide patients to provide themselves with the answers, as described by Chiara (professional nurse, 24 years old): *“What we do is to make them understand, by asking some questions that could help them to have a clear vision of their situation: ‘Do you remember why you have been to the emergency room?’ ‘Have you considered … have you noticed that, since you came here, things have gotten slowly worse and worse?’”.* However, often, not even this solution is fully satisfying: *“I am not happy after this, so I go out, and I feel bad, and I report this during our group encounters, and I express there all my discontent for the fact that I could not give a real answer”*, said Sara (professional nurse, 41 years old).

Patients realize that something is wrong as their clinical situation worsens, but since they do not obtain any proper explanations, their mood deteriorates, as reported by Alessia (professional nurse, 26 years old): *“The more he could not obtain an answer to his questions, the more his mood worsened, and consequently his health got worse too. He was restless”*. The lack of awareness *“often makes patients anxious, contrary to what the relatives believe, because they feel sick, suffer, and their bodies tell them that they are dying, while no one seems to be interested in this”*, explained Elena (professional nurse, 45 years old).

However, when patients become aware of the truth, their attitude toward nurses turns from begging into blaming, as described by Paola (professional nurse, 54 years old): *“They think we did something wrong, and that we are guilty. Insincerity is interpreted as concealment of errors and mistakes”*. Whereas the lack of sincere communication is perceived as very negative, sincere truth telling is associated with authentic and peaceful relationships, as reported by Elisa (nursing student, 23 years old): *“Everything becomes easier … Patients accept you inside their homes, they are more compliant and involved. They are grateful for all your interventions aimed at reducing their pain and suffering”*. Through this genuine communication, relationships are more satisfying and profound, according to Alessia: *“With the patients who are aware of their prognoses, I find myself talking about some important, serious matters that can help them but also enrich me. I can think of their existential narrations and learn more about the meaning of life”*.

### 3.2. Second Area of Thematic Prevalence: Relatives and the Conspiracy of Silence

A second relevant thematic category concerns the relationship with the patients’ relatives, who are often considered to be the most substantial obstacle to sincere communication. Indeed, there are several situations in which they openly request patients not be informed about their condition, as reported by Rosa: *“Relatives are often the biggest problem. They want to conceal everything from their dying beloved. We cannot do anything and we have to maintain the balance between doctors, family members, and patients without saying anything compromising”*. In the opinion of our participants, this situation is the direct consequence of the relationships between physicians and the families, which permit the avoidance of direct communication between doctors and patients, as highlighted by Marco (nursing student, 24 years old): *“The doctor speaks with the relatives in order to know what to say, what not to say or simply to assess the situation”*, and in this way, *“as relatives perceive that we are going to tell a patient the truth, they interfere, and their determination not to inform the patient destroys any information that may help the patient to understand”*, said Matteo (professional nurse, 44 years old). However, the problem derives from their inability to truly communicate with their beloved: *“Actually, they block any form of honest communication with their beloved because, in reality, they have not yet dealt with the reality. They do not want to face death, and therefore, they are not able to help their dying relatives”* (Stella); *“All this is the effect of the relatives’ selfishness. I see it this way, they are afraid to face the end of the story and prefer to pretend nothing is happening”* (Laura). In both nurses’ and students’ opinions: *“All this is very different from other countries, for example, the UK, where doctors must explain everything to their patients. I worked in England for a year and a half and the relatives there were not taken into consideration before the patient, no way! Absolutely impossible, especially if the patients were alert”* (Alessia).

However, collaboration with relatives is considered essential, since they know their beloved and their preferences, as Rosa highlighted: *“We do not think we can know our patients better than their relatives, even if they stay here for months. The family members know them better than anyone else”*. Then, nurses are trapped in a network of relational contradictions, and this causes intense frustration: *“From all this, very bad feelings spring, but you cannot say anything anyway because there is the relative who … controls you like a puppet”*, said Elisa.

In order to manage these situations, participants recognize the need to develop an educational approach towards the relatives so that they can better accept their loved one’s condition, given that, as Giovanni said: *“Patients’ relatives represent their best support during end-of-life care. The patient needs to be serene to cope with sickness and death, and the caregivers too should be able to cope with this crucial passage by taking care of the patient”*.

### 3.3. Third Area of Thematic Prevalence: Ethical Dilemmas and Emotional Burden

The difficulties emerging from the untruthful communication between doctors, relatives, and patients produce many ethical problems for nurses, as described by Erika (nursing student, 22 years old): *“Is it right to tell them the truth? Is it right that they enjoy the last ten days of their life without knowing that they are dying? Or rather, would it be better to tell them exactly what is happening and prepare them for the end? However, all these questions do not have an answer and you do not know what it is best to do”*. The bewilderment is also accentuated by identification with the patients, so that the resentment against relatives becomes unbearable, as reported by Stella: *“If I were the patient and they had not told me what illness I had, it would be … horrible, a horrible way to end my life, so I felt distressed, a bit angry … angry with those who decided to lie to him”*. The most disturbing thought to manage is their own perception of being part of the same conspiracy of silence originating from the doctor–relative relationship: *“I do not like to lie or tell the truth partially. I want to be sincere. When I am required to lie, I feel like I am betraying my principles”* (Alessia); *“All this is very unmanageable. It is problematic because I find it difficult to lie … I felt fake too, false, a liar really”* (Erika).

However, despite all this, nurses admit that, sometimes, there are patients who do not want to talk about their death: “Everyone can know a certain amount of truth, and we have to respect the individual’s needs to know or be ignorant of the truth” (Julia, professional nurse, 26 years old); “It is important that a patient should never be robbed of his/her hope. You must never take it away. We have seen people who were supposed to die in a week and instead lived for other 3–4 months, and even quite well, because they had hope” (Rosa). Even if both students and nurses agree that open communication is the best way to proceed, at the same time, they admit that they do not know how to manage the psychological consequences of breaking bad news in the correct way: “It is a terrible responsibility because, after such information, the patient’s life changes forever. When the time comes to tell the truth, everything is more difficult. […] The nurse who was on shift was not able to manage the crisis of the patient who had just received the bad news because he had hoped that there would still be some future for him”, said Sara. 

Most difficult to manage are the ethical dilemmas that arise when the patients’ knowledge is ambiguous. In such cases, it is impossible to answer their questions because they just want reassurances. Patients suspect that they are going to die, but actually hope that they are not: *“She knew she was sick but she did not want the details, but she looked for reassurance from the nurses”* (Valentina, nursing student, 21 years old); *“They very often know we do not tell them the truth, but they want to hear something different”* (Giada, professional nurse, 69 years old).

The tension between conspiracy of silence and ethical dilemmas causes an intense burden because participants believe that they are actually betraying their patients: *“You feel a bit like a betrayer because they should trust you, but you keep hiding things from them”* (Ester, nursing student, 22 years old); *“I return home, and I feel really guilty. I feel horrible”* (Eva, nursing student, 22 years old); *“All this frustrates me, because it appears to me that I am not doing my job properly”* (Violet, nursing student, 23 years old); *“You feel a nobody … helpless”* (Elisa); *“I should be a pivot for my patients, and if I can do nothing to make their journey better, I believe I failed as a nurse. I am a nurse who values myself less than I could”* (Marco). Resentment toward doctors and relatives is stressful as well, as reported by Matteo: *“Sometimes we would like to take it out on the relatives or on the doctors. It is very hard coming to the end with all these dilemmas. All this makes us angry”*. In order to deal with this burden, participants agreed that they really need support. They find it in psychologists and, more specifically, in sessions of group therapy, as described by Giorgia (professional nurse, 54 years old): *“Luckily, we here, in the hospice, have a psychologist who organizes some group sessions. Everyone expresses what he/she feels, his/her own experiences, and we go on like this”*.

### 3.4. Fourth Area of Thematic Prevalence: Absence of a Bridge Between Active Treatments and Palliative Care

In the participants’ opinions, all these difficulties derive from lack of a suitable transition from active treatments to palliative care, reflecting poor management by the healthcare system. Patients and families need time to accept bad news, from the diagnosis to the inauspicious prognosis, and giving information too late hinders their ability to accept the terminally ill’s condition: *“They arrive unprepared. They were told that this is not a hospice but a clinic, so their expectations are very high”* (Elia, professional nurse, 33 years old); *“They need time, and we need the same as well. We need time to get to know each other and form a good relationship. It is necessary to manage this passage accurately because the entire life of patients and their families is changing”* (Elia). If this passage is poorly accomplished, they develop unrealistic expectations and *“When the reality is finally undeniable, a terrible shock follows. Dealing with this in a hospice is very complicated, because when someone has been sick for years and nobody has ever told them the truth, it is quite impossible to recognize their psychological resources to inform them that there is no hope”* (Stella); *“We should start from there, making the doctors aware of this, because you know, any doctor just considers his/her own job, they do not see all the difficulties that follow their incapacity to manage the bad news”* (Giorgia).

In their opinion, “It is necessary to remove the general censorship of mortality, and it is important to make people aware of palliative care”. Most people “do not even know what a hospice is, not even their relatives. In Italy, we are unfortunately still really far from knowing what a hospice is”, according to Rosa. The taboo surrounding death also leads to the fact that not even the nurses really possess the proper instruments and training to manage their patients’ end-of-life care appropriately, as reported by Claudio (professional nurse, 50 years old): “We should first of all prepare ourselves. We should have some proper instruments to communicate with our patients, because unfortunately, the majority of us never had proper training, and you do this because you feel you can, or perhaps because you feel particularly inspired that day, and you say the right words, but maybe the day after that, in the same situation, you are taken aback. We lack proper training”.

## 4. Discussion

Lack of proper, sincere communication between healthcare professionals and patients, especially concerning the disclosure of bad news (truth-telling) is a well-recognized and complex issue, especially when a patient is terminally ill and still not fully aware of his/her condition [2,3].

The present research allowed the further exploration of the critical issues encountered by nurses who work in palliative care units when they have to deal with patients who are not aware of their diagnoses and/or prognoses. Since nurses represent the professional category that spends the most significant amount of time in direct contact with patients, and as a result of not being allowed to communicate any prognoses, they are usually those who also suffer most intensely from the impact of critical issues related to the lack of truth-telling [17].

Indeed, the participants of this study reported a strong emotional burden linked to these situations, with high levels of anxiety, fear of betraying the patient’s trust when doctors or family members demand their silence concerning the patient’s diagnosis/prognosis, and the feeling that they are actually jeopardizing their relationships with their patients. Nurses, moreover, in the absence of sincere communication with the patients, feel that they constantly struggle to offer them palliative care of a high quality, including proper assistance at the end of life, but they feel, at the same time, frustrated because of their inability to actually provide all of this. This often leads to a lack of satisfaction, sadness, demoralization, and a fear of not doing what is actually best for the patient [15,16].

Such a heavy burden largely stems from the ethical dilemmas constantly experienced by nurses because of the conflict between their desire to be sincere and the extreme difficulty, or impossibility, to do so, as reported by other research [11]. This often leads to feelings of uncertainty and confusion, but also frustration, helplessness, and guilt when nurses are required to lie or to evade their patients’ requests for information [16].

The core issue of ethical dilemmas in the medical field within the international context is the patients’ fundamental right to self-determination and consent [5]. The nurses’ frustration results, in particular, from the fact that even though the doctor decides whether to tell the truth or not, the nurses are the ones who must deal with the consequences of their decisions, along with their patients. 

Moreover, feelings of rage toward those (patients’ relatives or doctors) who impose on nurses their decision to not properly inform patients are also common [13]. Patients’ relatives are often considered to be the main obstacle to truth-telling practice, since it is very common for them to impose their choice of silence on nurses, most of the time because they desperately try to ignore the reality [9,14,15,30].

Other possible reasons that may obstruct truth-telling have been linked by the participants to the broader Italian cultural context that still does not accept death as an essential part of life and tends, therefore, to even refuse to talk about it, even inside palliative care units. This determines, in turn, a serious lack of proper training for healthcare professionals, who actually find themselves unprepared to deal with their patients’ end-of-life care. Since they have no professional skills to deal with these situations, and in order to protect themselves from the strong ethical dilemmas and confusion they experience, nurses report that they prefer to avoid patients who are unaware of their condition, even though this does not actually allow them to feel better about it [14].

**Figure 1:**
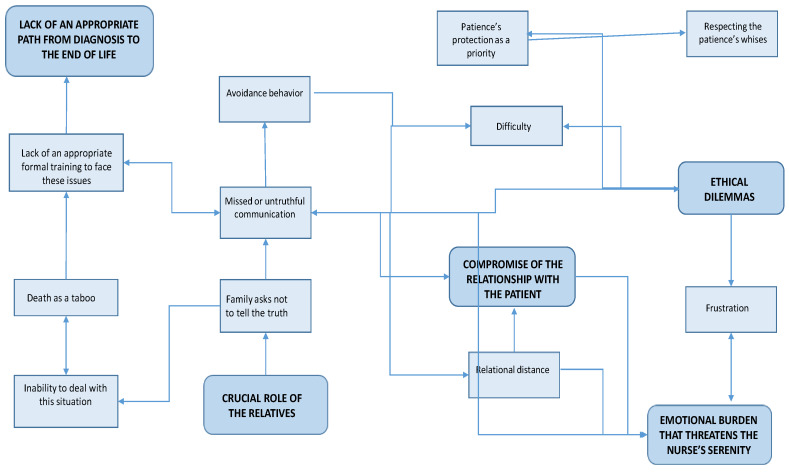
Main critical issues described by the participants.

However, some participants have invented a different, more functional way of dealing with the distress linked to truth-telling. It is a particular kind of communication with the patient that aims to help him/her understand his/her health condition through some questions posed by the nurse, which highlight the patient’s current condition and symptoms, without openly telling the patient that he/she is actually dying. Furthermore, participants also found that the support of psychologists helps them to deal with these paradoxical situations. In particular, they considered some group therapy sessions organized by a psychologist, in which they could share their experiences with all their colleagues, as comforting. A summary of the main themes that emerged from the participants’ narrations and of the relations between them is represented in Figure 1.

A significant change in the way the healthcare professionals communicate amongst themselves and their patients is the primary necessity. The participants’ narrations represent a complex challenge for all the parties involved; however, there is a conceptual framework from which we can begin to address it. At its core is the fundamental right of self-determination for patients who have decision-making capacity. One of the greatest difficulties identified by nurses in the present research is the obstacle to sincere communication imposed by patients’ family members. It is the right of patients to know as much about their medical condition as they wish to know and to make their own decisions about their care. At the same time, patients have a right to request that health providers not make them aware of their medical condition. In addition, patients should decide with whom to share their medical information and whether or not they wish to delegate decision-making to someone other than themselves even if they retain capacity for decision-making. The medical provider should elicit the patient’s preferences without family or friends present to avoid undue influence over their decisions. The expressed wishes of the patient should be documented in his/her medical record in order for them to be shared and honored by all members of the medical team. If the patient requests truthful communications, all members of the team, including nurses, would be allowed to engage in them. The judgement of physicians or family members about truth-telling must never substitute the patient’s own wishes.

This patient-centered approach would help prevent disagreements among different members of the healthcare team, a problem that has been highlighted by many nurses who find themselves in conflict with other colleagues, especially doctors, who might not share their view on the necessity to properly communicate with patients. The unmistakable will of the patient, clearly documented in his/her medical record, would guide communications and help put an end to these misunderstandings.

The patient’s inalienable right to choose freely for himself/herself is indeed stated by the law both in Italy (e.g., law n. 219 of 2017) and internationally (e.g., the Oviedo Convention of 1997). Hospitals, hospices, and other healthcare facilities and agencies should enshrine these principles into their own policies in order to set expectations of their staff to abide by the law and place patients at the center of medical practice. Institutional policies would help empower nurses and other healthcare providers to hold each other accountable. 

Psychologists can help providers develop the necessary skills to engage patients in difficult conversations about their preferences and goals of care. They can educate them on the proper way to explore a patient’s will to be informed (or not to be), communicate eventual bad news, and to deal with a patient’s emotional responses as well as their own. Psychologists could also help mediate complex interactions between the healthcare professionals’ needs on one hand and the patients’ and their families’ ones on the other, encouraging an open dialogue. 

In addition to healthcare providers, there is a need to educate society in general, which should also include Death Education [27], in order to be able to finally accept human vulnerability and death in our culture, to be able to discuss about it, and to make plans for future medical treatments serenely. 

## 5. Conclusions

This research allowed some fundamental issues that are still critical in the relationship between nurses and patients to be highlighted, especially concerning sincere communication of the patient’s diagnosis and prognosis (truth-telling).

This is a particularly delicate issue, since nurses are not allowed to give a patient information concerning his/her condition without a doctor’s approval, and, even though the importance of sincere communication with a patient is now generally well recognized, doctors still very often opt for incomplete or totally absent sharing of information, most of the time because they lack the psychological skills needed to manage these situations, and because it is very common for family members to ask a doctor not to tell their relative the truth.

Since this important issue can actually be considered part of a widespread conspiracy of silence (even inside healthcare contexts), the main element on which it would be fundamental to take action is the professional curricula useful in understanding and approaching death and dying in health profession degrees. Considering what has emerged from this research, it appears fundamental to adopt a completely new attitude toward death and dying, especially while dealing with terminally ill patients. This will obviously require a broader cultural change, so death itself will not be considered as something to ignore, but accepted as part of life itself, something to talk about, and a crucial theme for every healthcare professional’s training. The starting point is for health professionals to respect and honor the patient’s right to self-determination within a health system focused on providing patient-centered care. 

### Limits of the Research

The main limit of this research is linked with its participants, since it involved only the nurses’ points of view, and, in relation to this aspect, it would also be very interesting to investigate the opinions and thoughts of the other figures involved in this matter.

It would be very important to explore doctors’ opinions, as they are, in fact, those who generally decide not to tell a patient the truth, and to learn about the opinions of psychologists, who support doctors and nurses, as well as patients and relatives in their end-of-life journeys. Of course, caregivers’ opinions need to be studied too, since it is they who often ask doctors and nurses not to disclose a poor diagnosis and/or prognosis to their loved ones, and who know the patients more intimately. Moreover, since nurses who work in palliative care are prone to burnout [31] and depression [32], future research is required in order to explore the link between burnout, depression, and truth-telling among nurses in palliative care.

## Figures and Tables

**Table 1:** Participants: Nurses and students.

Number of Participants		Gender		Age		Length of Service (in Months)		Months of Experience in Palliative Care	
Professionals	Students	Male	Female	Mean age		Under 20	Over 20	Under 5	Over 5
				Professionals	Students				
25 (53.2%)	22 (46.8%)	9 (19.15%)	38 (80.85%)	39 years	23 years	17 (68%)	8 (32%)	14 (56%)	11 (44%)
				Standard Deviation:		Standard Deviation:		Standard Deviation:	
				13.7	1.36	151.60		61.50	
				Total Standard Deviation: 12.90		Mean (in years): 13		Mean (in years): 5	

## Data Sharing and Accessibility

The data that support the findings of this study are available on request from the corresponding author. The data are not publicly available due to privacy or ethical restrictions.
